# WX-132-18B, a novel microtubule inhibitor, exhibits promising anti-tumor effects

**DOI:** 10.18632/oncotarget.17710

**Published:** 2017-05-09

**Authors:** Fang Guan, Rui Ding, Qi Zhang, Wei Chen, Feifei Li, Long Long, Wei Li, Linna Li, Dexuan Yang, Lan Xie, Shoujun Yuan, Lili Wang

**Affiliations:** ^1^ Beijing Institute of Pharmacology and Toxicology, Beijing, 100850, China; ^2^ State Key Laboratory of Toxicology and Medical Countermeasures, Beijing, 100850, China; ^3^ Beijing Institute of Radiation Medicine, Beijing, 100850, China

**Keywords:** tubulin inhibitor, anti-tumor effects, high content assay, cellular phenotype, colchicine-binding site

## Abstract

Cancer drug researchers have been seeking microtubule-inhibiting agents (MIAs) with higher bioactivity and lower toxicity than currently marketed drugs. WX-132-18B, a novel structural compound synthesized at our institute, specifically bound to the colchicine-binding site on tubulin rather than the vinblastine site, and concentration-dependently reduced microtubule content via depolymerization. It exhibited the same cellular phenotypic profiles as the classic MIAs (colchicine, vincristine, and taxol), including inducing cell cycle arrest at the G2/M phase, triggering tumor cell apoptosis, promoting nuclear membrane permeability, reducing mitochondrial membrane potential, and disrupting the redox system balance. Importantly, WX-132-18B displayed more potent *in vitro* bioactivity (IC_50_ 0.45–0.99 nM) than did the classic MIAs; it inhibited the proliferation of human umbilical vein endothelial cells and seven types of human tumor cells, especially the taxol-resistant breast cancer cells MX-1/T. WX-132-18B also dose-dependently inhibited mice sarcoma, human lung, and gastric cancer xenograft tumors and the formation of tumor blood vessels in mice. In conclusion, WX-132-18B is a novel microtubule-depolymerizing agent that selectively acts on the colchicine-binding site of tubulin and exerts potent *in vitro* and *in vivo* anti-tumor effects. These characteristics, along with its anti-angiogenesis and anti-drug resistance properties, make WX-132-18B a promising anti-tumor drug candidate.

## INTRODUCTION

**Figure 1:**
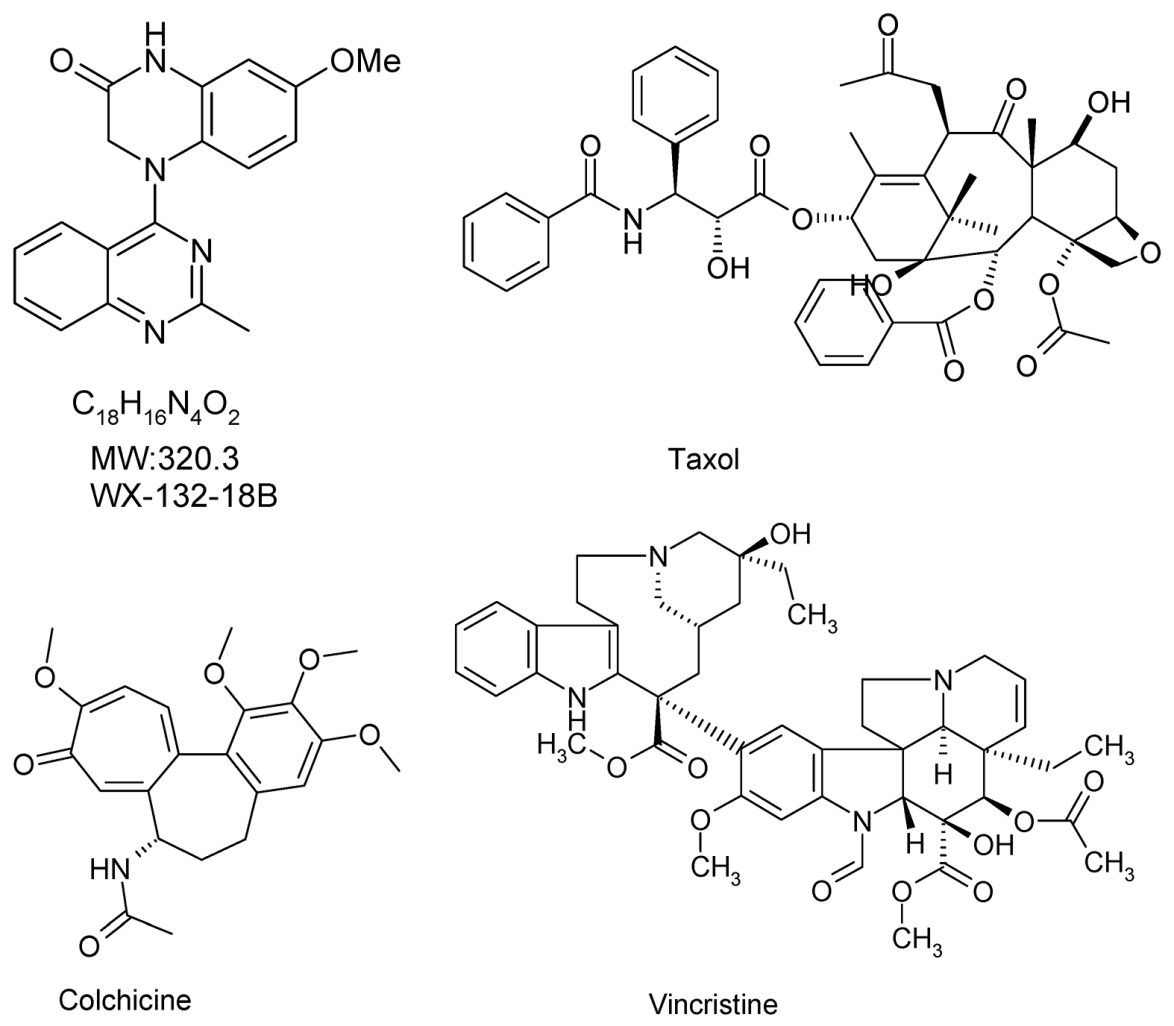
Chemical structure of compound WX-132-18B and three known microtubule-inhibiting agents (taxol, colchicine, and vincristine)

Microtubules are essential components of the cytoskeleton and are involved in many cellular processes including cell morphology maintenance, intracellular transport, and mitosis [1, 2]. The dynamic cycle between tubulin and microtubules is critical for cellular function, especially in normal mitosis [3]. Microtubule inhibition is therefore a potential strategy for cancer treatment, because inhibiting microtubule dynamics would arrest the cell cycle and thus inhibit tumor cell proliferation [4]. Three well-known microtubule-inhibiting agents (MIAs) with different tubulin binding sites are widely used for the treatment of various types of cancer and gouty arthritis: the microtubule depolymerizing drugs colchicine and vincristine, and the microtubule polymerizing agent taxol (Figure 1) [5, 6]. Taxol and vincristine are first-line treatments for lung cancer [7], breast cancer [8], and ovarian cancer [9]. As is seen with other anti-cancer drugs, intolerable toxicities [10] and drug resistance restrict the clinical use of these drugs. Poor water-solubility and shortage of raw material also limit clinical use of taxol. The development of novel microtubule inhibitors with higher activity, lower toxicity, and better anti-drug resistance than currently available options remains an urgent need and a current research focus [11, 12]. Combretastatin A4, a colchicine site-targeted compound and vascular-disrupting agent that could destroy tumor blood vessels and effectively inhibit solid tumors recently passed Phase I and II clinical trials [13–15]. In addition, MIAs with high cytotoxic activity currently are also the first choice for antibody-drug conjugate [16, 17].

WX-132-18B is a new structural compound synthesized at Beijing Institute of Pharmacology and Toxicology (Figure 1). Preliminary screening indicated that WX-132-18B targets microtubules and displays high bioactivity in inhibiting tumor cell proliferation. In this study, the anti-tumor effects of WX-132-18B were first examined in a series of human tumor cell lines. Three classic MIAs (colchicine, vincristine, and taxol) were used as positive controls. The target of WX-132-18B was thoroughly validated via the tubulin competitive binding test, cellular skeleton multiparametric high content assay (HCA), and microtubule inhibition-related cellular functional assays. WX-132-18B was also screened for hepatotoxic phenotype profiles and 22 tumor-related signaling pathways. Finally, the *in vivo* anti-tumor effects of WX-132-18B were systematically evaluated in three different xenograft tumor mouse models.

## RESULTS

### WX-132-18B inhibited tumor cell proliferation

**Figure 2:**
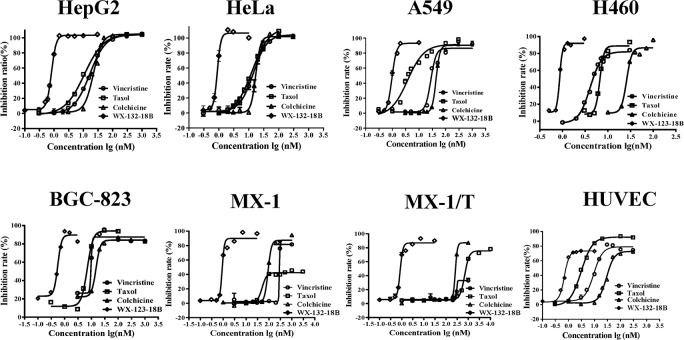
Anti-proliferation effects of compound WX-132-18B on HepG2, HeLa, A549, H460, BGC-823, MX-1, MX-1/T, and human umbilical vein endothelial cells Cells were treated with different compounds for 72 h and then assayed with the sulforhodamine B method as indicated. Values are mean±SD, n=3.

The anti-tumor bioactivity of WX-132-18B was evaluated using the sulforhodamine B (SRB) method in cancer cell lines HepG2, HeLa, A549, H460, BGC-823, MX-1, taxol-resistant breast cancer cells MX-1/T, and human umbilical vein endothelial cells (HUVECs). As shown in Table 1, WX-132-18B exhibited the strongest inhibitory activity on all the tested cell lines compared to the three control MIAs (taxol, colchicine, and vincristine) tested. The control MIAs showed evident cellular selectivity and had a lower IC_50_ value on HUVECs and a much higher IC_50_ value on MX-1/T than their effects on the other cancer cell lines. However, WX-132-18B did not show selectivity in any tested cancer cell line and exhibited more potent inhibition activity than the three known MIAs on all cell lines, with an IC_50_ value less than 1 nM. Similarity in the shape of the concentration-inhibition curve between WX-132-18B and colchicine suggests that they may have similar mechanisms of action (Figure 2).

**Table 1:** IC_50_ values of the tested compounds in different cell lines

	IC_50_ (nM)							
	HepG2	HeLa	A549	H460	BGC-823	MX-1	MX-1/T	HUVEC
Taxol	10.68±0.61	12.86±0.25	4.81±0.61	6.7±0.21	6.59±0.76	50.03±8.17	689.76±25.08	3.04±0.12
Colchicine	21.17±1.22	14.19±0.53	43.80±1.64	26.74±2.26	12.24±3.61	90.70±4.74	260.48±5.92	27.67±1.79
Vincristine	16.51±0.36	16.76±0.33	27.80±2.75	4.87±0.34	6.03±1.64	279.35	302.77	9.15±0.78
WX-132-18B	0.80±0.02	0.90±0.01	0.99±0.06	0.86±0.03	0.45±0.07	0.90±0.04	0.84±0.04	0.70±0.04

Cells were treated with different compounds for 72 h. Values are mean±SD, n=3

### WX-132-18B induced microtubule depolymerization in A549 cells

**Figure 3:**
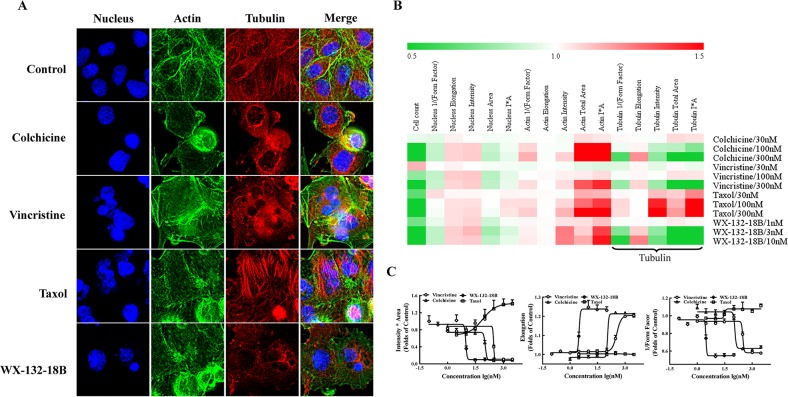
Impact of compound WX-132-18B on cellular skeleton and nucleus **(A)** Representative images of nucleus, actin, and tubulin of A549 cells after treatment with vehicle (0.1% dimethyl sulfoxide), colchicine (100 nM), vincristine (100 nM), taxol (100 nM), or WX-132-18B (3 nM) for 24 h. Tubulin was visualized with an anti-α-tubulin antibody (red), actin was visualized with Alexa Flour 488 phalloidin (green), and cell nucleus was visualized with Hoechst 33342 (blue). Images were acquired with an IN Cell Analyzer 1000 using a 20× objective lens. **(B)** Heat map analysis of tested compounds on cellular morphology multi-parameter assay in A549 cells by high content assay. **(C)** Concentration-effect curves of tested compounds on three different parameters of tubulin in A549 cells. Values are mean±SD, n=3.

Because MIAs are commonly used to treat non-small cell lung cancer, the effects of WX-132-18B on microtubules were examined using cytoskeleton multiparametric HCA in A549 cells. As shown in Figure 3A, while taxol promoted microtubule aggregation, cellular microtubules were significantly disrupted and reduced after 24 h treatment with WX-132-18B, colchicine, and vincristine. The impacts of the tested drugs on the cytoskeleton are illustrated in a heat map (Figure 3B) showing fold-changes of cytoskeleton parameters including F-actin, tubulin, and nucleus, following treatment with various concentrations of the tested compounds. WX-132-18B, colchicine, and vincristine present similar cellular phenotype profiles, whereas their effects on tubulin-associated parameters are different from those exhibited by taxol. Furthermore, the concentration-effect curves shown in Figure 3C indicate that WX-132-18B, colchicine, and vincristine reduced tubulin I*A and 1/(Form Factor) and increased tubulin elongation in a concentration-dependent manner. Tubulin I*A, 1/(Form Factor), elongation indicating the average cellular content of tubulin, the mean roundness index of tubulin, and the mean ratio of the short axis to the long axis of tubulin, respectively (as shown in Table 2). WX-132-18B was more potent than the known MIAs at low concentrations; the EC_50_ of WX-132-18B is 9.43, 2.99, and 3.12 nM on these three different tubulin-related parameters (Table 3). These results indicated that, similar to the depolymerizing agents colchicine and vincristine, WX-132-18B also reduced tubulin content and shortened or broke down microtubules. Taxol, in comparison, increased tubulin content in a concentration-dependent manner, but had little or no effect on 1/(Form Factor) and elongation parameters. Time-effect observation demonstrated that the MIAs and WX-132-18B showed interference with the microtubule structure at 6 h; while taxol promoted microtubule aggregation, WX-132-18B, colchicine and vincristine augmented microtubule degradation (Supplementary Figure 1). These results indicate that WX-132-18B is a potent microtubule-depolymerizing agent rather than a microtubule-stabilizing agent.

**Table 2:** Multi-parametric cellular phenotypic assay panel

Assay targets	Parameters	Implications
Assay1		
Nucleus	Cell count	Number of cells
	Area	Area of the identified nucleus
	Intensity	Mean nuclear intensity
	Elongation	Ratio of the short axis to the long axis of the nucleus
	1/(form factor)	Mean roundness index of the nucleus, perimeter ^2^/(4π × area)
Actin	Intensity	Average intensity of pixels for the filaments
	Total area	Total area of filaments
	I*A	Average content of actin in cells
	Elongation	Mean ratio of the short axis to the long axis of the filament
	1/(form factor)	Mean roundness index of actins, perimeter ^2^/(4π × area)
Tubulin	Intensity	Average intensity of pixels for tubulin
	Total area	Total area of tubulin
	I*A	Average content of tubulin in cells
	Elongation	Mean ratio of the short axis to the long axis of tubulin
	1/(form factor)	Mean roundness index of tubulin, perimeter ^2^/(4π × area)
Assay2		
NMP	Cell intensity	Average pixel intensity in the cell region immediately adjacent to TOTO-3
	Intensity	Average intensity of TOTO-3 pixels within the nucleus
	(Nuc-Background)Int	Intensity of subtracting Background Intensity from Nucleus Intensity.
MnSOD	Count	Number of MnSOD attributed to the cell
	Spacing	Measure of the inter-MnSOD distance
	Distance to Nuc	Mean distance from the MnSOD center of gravity to the nucleus center of gravity
	Total area	Total area of MnSOD attributed to the cell
	Intensity	Average intensity of pixels within MnSOD
MMP	Count	Number of mitochondria attributed to the cell
	Spacing	Measure of the inter-mitochondria distance
	Distance to Nuc	Mean distance from the mitochondria center of gravity to the nucleus center of gravity
	Total area	Total area of mitochondria attributed to the cell
	Intensity	Average intensity of pixels within mitochondria
Assay3		
Annexin V	Intensity × total area	Early apoptosis
Annexin V+PI	Intensity × total area	Late apoptosis
PI	Intensity × total area	Necrosis

Output parameters and their implications derived from IN Cell Analyzer Workstation 3.5 and the Multi Target Analysis module for high content screening (GE HealthCare, USA). Nuclear analysis was included in each assay.

**Table 3:** EC_50_ values of testing compounds on tubulin parameters in A549 cells

	EC_50_ (nM)		
	Intensity*Area	Elongation	1/(Form Factor)
Taxol	75.84±6.73	-	30.89
Colchicine	105.5	111.62	114.1
Vincristine	293.2	352.03±18.10	30.89
WX-132-18B	9.43	3.12	2.99

Cells were treated with different compounds for 24 h. Values are mean±SD, n=3

### WX-132-18B bound to colchicine-binding site on tubulin

**Figure 4:**
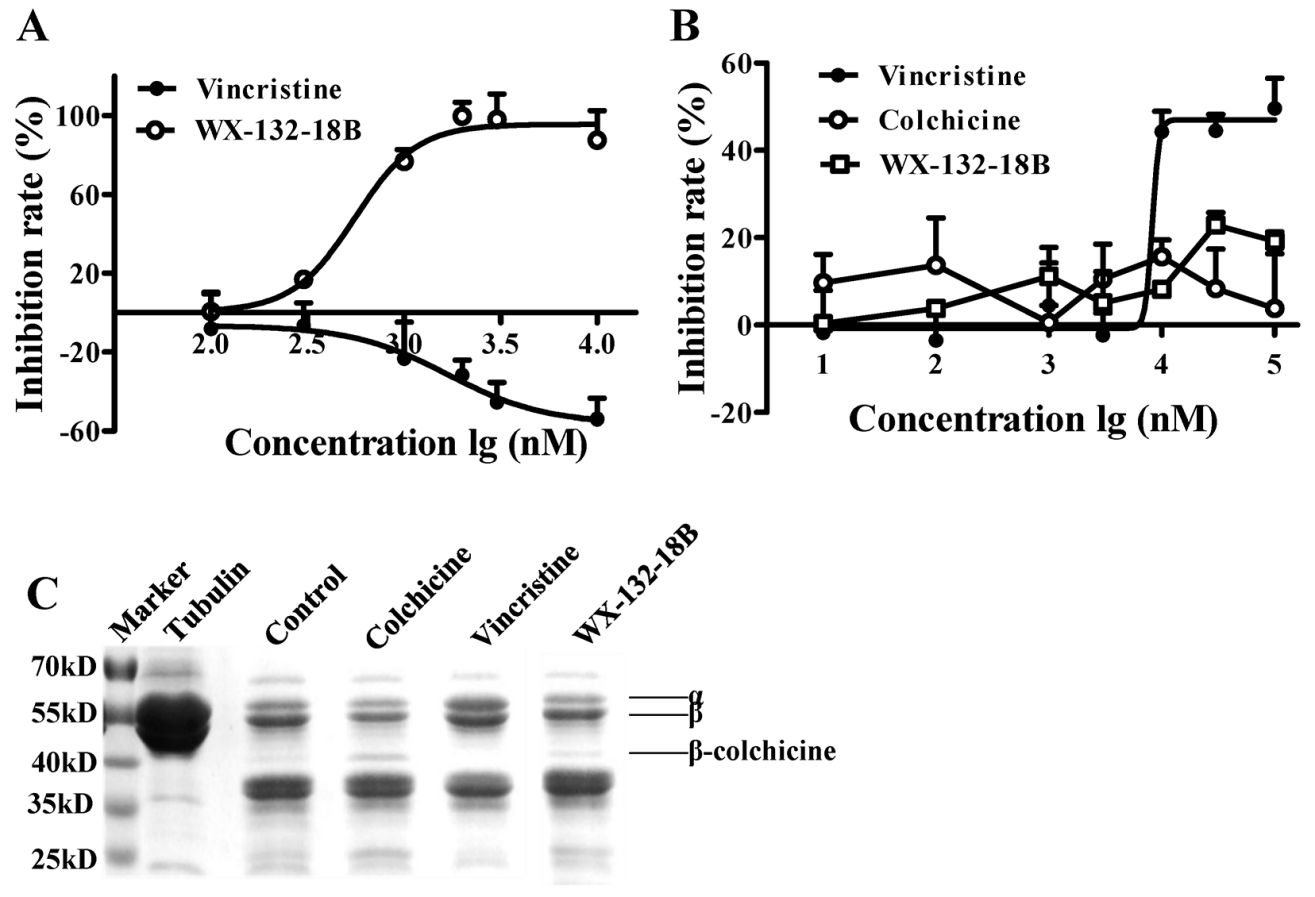
Binding site assay of compound WX-132-18B on tubulin **(A)** WX-132-18B competitively inhibited the binding of colchicine to tubulin. The inhibition rate is expressed as the percentage (%) of decreased fluorescence of the colchicine-tubulin complex. **(B)** The impact of compounds on the binding of BODIPY FL-vinblastine-tubulin complex. Values are mean±SD, n=3. **(C)** Limited proteolysis assay. Tubulin was digested with trypsin following preincubation with vehicle (0.1% dimethyl sulfoxide), 10 μM of colchicine, vincristine, and WX-132-18B. Tubulin treated with WX-132-18B showed patterns of proteolysis similar to those of colchicine.

Given that most tubulin depolymerizing agents bind to either colchicine- or vinblastine-binding sites [18], the binding characteristics of WX-132-18B were examined on these two sites on tubulin via competitive binding assays on the molecular level. The increased intrinsic fluorescence produced by colchicine upon its binding to tubulin [19] was used as an indicator for WX-132-18B competition with colchicine in the tubulin binding assay. As expected, vincristine, a typical vinblastine-binding site drug, did not affect the fluorescence intensity of the colchicine-tubulin complex (Figure 4A). WX-132-18B, however, reduced the fluorescence intensity in a concentration-dependent manner, with an IC_50_ value of 0.47±0.10 μM.

Similarly, a fluorescent analog of vinblastine, BODIPY FL-vinblastine, was used in a vinblastine-binding site assay. As shown in Figure 4B, the fluorescence intensity of the BODIPY FL-vinblastine-tubulin complex was decreased in a dose-dependent manner by vincristine, with an IC_50_ value of 6.97±1.61 μM, but not by colchicine or WX-132-18B. WX-132-18B only slightly inhibited the fluorescence intensity of BODIPY FL-vinblastine-tubulin complex at a higher concentration (30 μM). The inhibition curve of WX-132-18B was similar to that of colchicine, but different from that of vincristine, indicating that WX-132-18B did not act on the vinblastine-binding site of tubulin.

Colchicine and vincristine bind to tubulin at different sites, which could specifically affect the enzymatic hydrolysates of tubulin [20]. To further confirm the tubulin binding characteristics of WX-132-18B, WX-132-18B-, colchicine-, or vincristine-tubulin complexes were digested using tosyl-phenylalanine chloromethyl-ketone (TPCK)-treated trypsin and resolved with SDS-PAGE electrophoresis. Figure 4C shows that the electrophoresis band spectrum of WX-132-18B is similar to that of colchicine and dissimilar to that of vincristine. WX-132-18B and colchicine both present an enhanced β-colchicine band, with more clear bands at the low molecular weight area. The β-colchicine band has been reported as a colchicine-site-related cleavage product produced by trypsin [21, 22]. The vincristine-tubulin complex did not display the β-colchicine band. These results reveal that WX-132-18B binds to the colchicine-binding site, rather than the vinblastine-binding site, of tubulin.

### WX-132-18B arrested cell cycle at the G2/M phase in A549 cells

**Figure 5:**
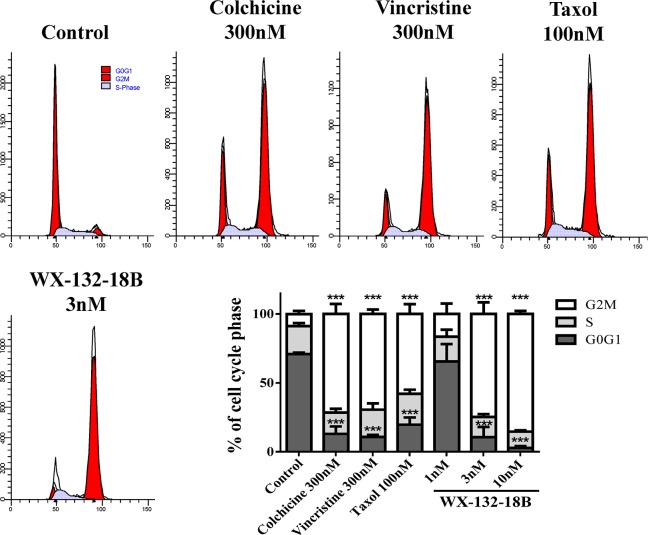
The impact of compound WX-132-18B on cell cycle of A549 cells A549 cells were treated with vehicle (0.1% dimethyl sulfoxide), colchicine (300 nM), vincristine (300 nM), taxol (100 nM), or WX-132-18B (3 nM) for 24 h and then assayed via flow cytometry. The bar chart shows the percentage of cell cycle distribution based on flow cytometry results. Values are mean±SD, n=3. *P<0.05, **P<0.01, ***P<0.001, compared with control group.

MIAs suppress the dynamics of microtubules, arrest the cell cycle in metaphase, and block cell division through misdirecting the formation of functional mitotic spindles in fast-dividing tumor cells [23]. As reported, the flow cytometry (FCM) assay confirmed that taxol (100 nM), colchicine (300 nM), and vincristine (300 nM) induced considerable G2/M phase arrest (Figure 5). WX-132-18B also arrested the cell cycle at the G2/M phase in a concentration-dependent manner. The potency observed with 3 nM WX-132-18B is similar to that observed with 100 nM taxol, 300 nM colchicine, and 300 nM vincristine, and the proportion of cells treated with 3 nM WX-132-18B in the G2/M phase was 74.77±8.47%, while that of the control group was only 8.68±2.30%. These data clearly indicate that WX-132-18B is a potent mitotic blocker.

### WX-132-18B induced apoptosis in A549 cells

**Figure 6:**
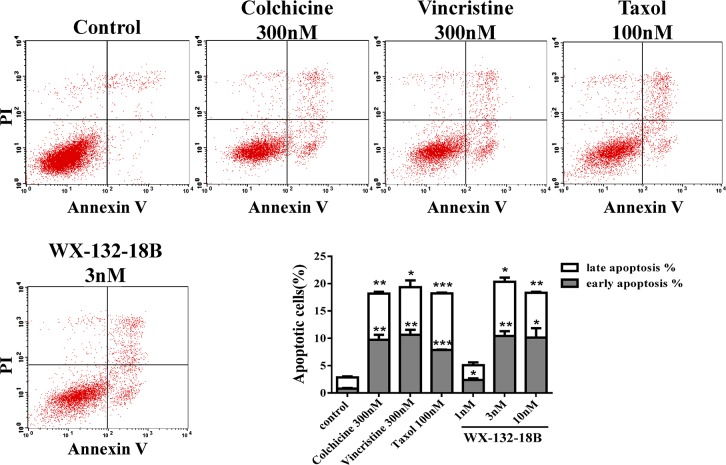
The impact of compound WX-132-18B on apoptosis in A549 cells A549 cells were treated with 300 nM colchicine, 300 nM vincristine, 100 nM taxol, or 3 nM WX-132-18B for 48 h and then assayed with flow cytometry. The bar graph shows a significant increase in the apoptosis rate of A549 cells. Values are mean±SD, n=3. *P<0.05, **P<0.01, ***P<0.001, compared with control group.

MIAs can induce apoptosis in tumor cells through several mechanisms, such as blocking the cell cycle [4], inhibiting the phosphorylation of Bcl-2 and Bcl-xl [24], activating caspases [11], upregulating E2F1 [25], and causing the release of cytochrome c [26]. The impact of WX-132-18B on cell apoptosis was examined in A549 cells by FCM with the Annexin V-FITC/PI dual-labeling method [27]. As shown in Figure 6, the three known MIAs induced obvious cell apoptosis after 48 h treatment in A549 cells. Similarly, WX-132-18B induced A549 cell apoptosis in a concentration-dependent manner. The effect of WX-132-18B at 3 nM is the same as that of the positive control drugs, as shown in the bar chart in Figure 6. Cocktail staining using Annexin V/PI/Hoechst 33342 combined with fluorescence imaging HCA showed similar results with FCM (Supplementary Figure 2).

### Effects of WX-132-18B on cytotoxicity in HepG2 cells

**Figure 7:**
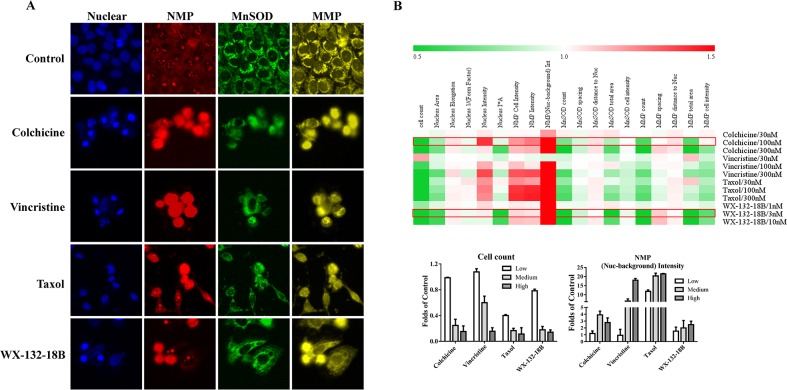
Effect of compound WX-132-18B on cytotoxicity in HepG2 cells **(A)** Representative images of NMP, MnSOD, and MMP in HepG2 cells treated with vehicle, colchicine (300 nM), vincristine (nM), taxol (100 nM) and WX-132-18B (3 nM) for 24 h. **(B)** Heat map analysis of tested compounds on cytotoxicity profiles in HepG2 cells; the bar chart shows the changes in parameter cell count and (Nuclear-background) Intensity of NMP more specifically. NMP, Nuclear membrane permeability; MnSOD, manganese superoxide dismutase; MMP, mitochondrial membrane potential. See Table 2 for meaning of the parameters.

Nuclear membrane permeability (NMP), mito-chondrial membrane potential (MMP), and manganese superoxide dismutase (MnSOD) are commonly used indicators of cytotoxicity [28]. Increase and decrease in MnSOD occur in response to oxidative stress and redox system damage, respectively. These parameters were compared among the three known MIAs and WX-132-18B in HepG2 cells, a cell line commonly used in hepatotoxicity screening. As shown in Figure 7, similar to the known MIAs, with the decrease of cell counts and the nuclear size in the concentration-dependent manner, the main parameters of MnSOD and MMP showed a concentration-dependent reduction, while NMP increased after HepG2 cells were treated with WX-132-18B for 24 h. The cytotoxic profiles of 300 nM colchicine, 100 nM taxol, 300 nM vincristine, and 3 nM WX-132-18B were similar. The effect of WX-132-18B and colchicine on the reduction of MnSOD and MMP is more potent than that of taxol or vincristine, and the maximum effect of WX-132-18B or colchicine on NMP is much lower than that observed for taxol or vincristine. These results indicate that WX-132-18B has a similar hepatotoxic characteristic and mechanism to known MIAs, and most closely resembles colchicine.

### Effects of WX-132-18B on cellular signaling pathways

To probe other potential mechanisms and exclude the off-target effects of WX-132-18B, 22 cellular signaling pathways (Supplementary Table 1) that are directly or indirectly related to tumor occurrence and development were further screened via HCA using pathway reporter cell lines. The four drugs were tested at concentrations of 0.03, 0.1, 0.3, 1 and 3 μM, which covers the pharmacodynamic and cytotoxic range for these molecules. The screening results indicate that, besides slightly inducing the formation of Rad51 foci (a DNA damage indicator) at higher concentrations (Supplementary Figure 3), WX-132-18B and the other three MIAs (taxol, colchicine, and vincristine) show no significant effect on the other 21 screened signaling pathways. This DNA damage effect revealed by the assay of Rad51 foci formation might indicate MIA toxicity, because the concentration at which it was observed was much higher than the microtubule-inhibiting concentration, and the effect observed is much lower than that observed with camptothecin, a drug used as a positive control to assess this pathway. Our pathway panel assay thus further demonstrates that, like the known MIAs, WX-132-18B has no impact on other screened signaling pathways.

### WX-132-18B strongly inhibited tumor growth *in vivo*

**Figure 8:**
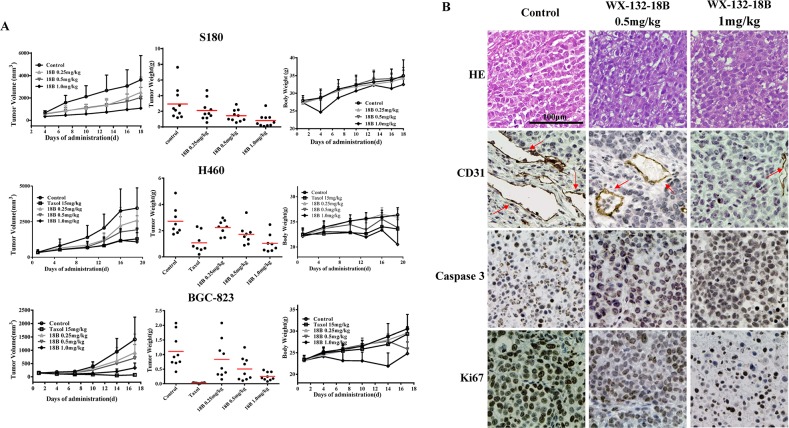
WX-132-18B inhibited tumor growth *in vivo* **(A)** Effects of tumor growth inhibition of WX-132-18B in S180 xenograft KM mice, H460 xenograft and BGC-823 xenograft Nu/Nu nude mice. Mice were treated with taxol or different doses (0.25, 0.5 and 1.0 mg/kg) of WX-132-18B for three weeks. Tumor volumes were measured before treatment and at the indicated days of treatment. At the end of the experiment, tumors were resected and weighed. Values are means±SD of tumor volume or weight for all the tested mice in each group. **(B)** Histological examination and immunohistochemical analysis of the expression and distribution of CD31, caspase 3, and Ki67 in BGC-823 xenograft tumor. The red arrow indicates blood vessels. Scale bar, 100 μm.

Because WX-132-18B showed high and non-selective anti-tumor activity *in vitro*, we first estimated the *in vivo* anti-tumor activity of WX-132-18B in S180 xenograft Kunming (KM) mice. The reason why we chose S180 is that mice xenotransplant model of sarcoma S180 cells is easy to establish and thus it is appropriate to be used in the primary evaluation of drug antitumor effect. Considering that non-small lung cancer is the principal indication of MIAs, and that xenotransplantation of A549 cells is not possible in mice, systematic bioactivity was evaluated in H460 human non-small lung cancer-bearing nude mice. Further, to disclose the underlying anti-tumor mechanisms of WX-132-18B, the effects on BGC-823 human gastric cancer-bearing nude mice were also examined, with taxol, the most potent MIA, as a positive control. Figure 8 shows that the S180 xenograft tumor grows exponentially after transplant; H460 and BGC-823 xenograft tumors displayed a slow increase in the early phase (from day 0 to day 10), and then the tumors expanded robustly (from day 10 to the end of the experiment). WX-132-18B treatment inhibited the growth of xenograft tumors in all three xenograft models in a dose-dependent manner. Neither mortality nor significant body weight loss was found during treatment with WX-132-18B, indicating that WX-132-18B is well tolerated and safe at the doses tested. In S180 xenograft KM mice, 1 mg/kg WX-132-18B resulted in a 70.06% (*P*<0.01) and 72.62% (*P*<0.01) inhibition on tumor volume and tumor weight, respectively. The antitumor effect of the 1 mg/kg dose of WX-132-18B is comparable to that of 15 mg/kg taxol in H460 xenograft and BGC-823 xenograft Nu/Nu nude mice. At the end of treatment, the inhibition rates of WX-132-18B on tumor volume and weight for the H460 tumor were 68.70% (*P*<0.01) and 61.90% (*P*<0.01), and in the BGC-823 xenograft tumor they were 76.06% (*P*<0.01) and 77.32% (*P*<0.01), respectively (Figure 8A). These data clearly suggest that WX-132-18B has potent anti-tumor activity *in vivo*.

To further disclose the underlying anti-tumor mechanisms of WX-132-18B, histopathological tests and immunoassays of cell proliferation, apoptosis, and angiogenesis markers were performed on tumor samples. Hematoxylin and eosin (HE) staining performed on BGC-823 xenograft tumor tissues (Figure 8B) showed that cellular nuclei in the untreated group were enlarged and deeply stained; however, the nuclei were shrunken, and the structure became incomplete after treatment with WX-132-18B. Furthermore, the expression of caspase 3 (indicator of cell apoptosis), Ki67 (indicator of cell proliferation), and CD31 (marker of angiogenesis) in the tumor tissue were observed by immunohistochemistry (IHC) assay. As shown in Figure 8B, WX-132-18B treatment dramatically increased the brown-stained caspase 3 and decreased the expression of Ki67. CD31, specifically expressed in vascular endothelial cells, was also reduced by WX-132-18B in a dose-dependent manner. The *in vivo* data further reveal that WX-132-18B inhibit tumor growth by inhibiting cell proliferation, inducing apoptosis, suppressing tumor angiogenesis, and destroying blood vessels.

## DISCUSSION

MIAs are commonly used as anticancer agents. High activity, anti-drug resistance and tumor angiogenesis inhibition are the focus of research aimed at the development of new MIAs [14, 29]. WX-132-18B, a novel structural compound designed and synthesized at our institute, showed broad-spectrum *in vitro* anti-proliferative activity in multiple human cancer cell lines, including taxol-resistant tumor cells, through specifically binding to the colchicine-binding site and concentration-dependently reducing the microtubule content. Further study revealed that WX-132-18B exhibited cellular phenotypic profiles similar to those of the classic MIAs, including inducing cell cycle arrest at the G2/M phase, triggering tumor cell apoptosis, promoting NMP, and reducing MMP and MnSOD, with no effect on 22 other tumor-associated signaling pathways we screened. More importantly, the *in vivo* pharmacodynamics study confirmed that WX-132-18B could does-dependently inhibit mice sarcoma, human lung, and gastric cancer xenograft tumors, as well as tumor blood vessels, in mice.

It is generally acknowledged that molecules with similar mechanisms of action usually exhibit similar biological activities [30, 31]. To determine the mechanism of action of WX-132-18B, three marketed MIAs with definite and representative binding sites were selected as references, and *in vitro* comparative studies were performed. MIAs interfere with the dynamic process of microtubules by inhibiting microtubule depolymerization or by hindering tubulin assembly through binding to the molecule. High content image (HCI)-based cellular skeleton multiparametric assay directly and quantitatively reflects the effects of tested drugs on the cellular skeleton, including microtubules, microfilaments, and nuclei simultaneously. This assay clearly indicated that the cellular skeleton phenotypic profile associated with WX-132-18B was similar to that associated with the microtubule-depolymerizing agents colchicine and vincristine, but dissimilar to that associated with taxol, the microtubule-polymerizing agent, under same cytotoxic conditions. The only cellular phenotype parameters that are different are those related to microtubule. WX-132-18B induced microtubule debris and reduced cellular microtubule content. The tubulin competitive binding tests and SDS electrophoresis of tubulin enzymatic hydrolysis products further revealed that WX-132-18B selectively bound to the colchicine site on tubulin. This is consistent with the result from the inhibition of tubulin polymerizing assay [32]. WX-132-18B was therefore demonstrated to be a microtubule-depolymerizing agent that binds to the tubulin colchicine site.

Both the microtubule polymerizing and depolymerizing agents interrupted microtubule dynamics, inhibited the formation of spindles, disrupted cell division from middle stage to late stage, downregulated cyclin, and arrested the cell cycle at the G2/M phase [23]. The compounds also phosphorylated Bcl-2 and Bcl-xL, activated caspases, promoted the release of cytochrome c [24, 26], and induced apoptosis [2, 23]. Therefore, G2/M phase arrest and apoptosis induction have been accepted as the major cellular effects of MIAs. In the current study, the quantitative analysis of FCM and HCA together disclosed that WX-132-18 possessed the same effects on cell cycle arrest and apoptosis induction as the three known MIAs.

Hepatocellular toxicity assessments play important roles in drug discovery and toxicology [33, 34]. To some extent, the cytotoxic profile can predict the underlying mechanism of the tested molecules. NMP, MnSOD, and MMP reflect cellular functional status in the nuclear membrane structure, redox systems, and mitochondria, respectively, and are commonly used as indicators of cytotoxicity and the mechanism of action. In this study, we examined and compared the cytotoxic effects of WX-132-18B and the three MIAs in HepG2 cells through multiparametric HCA of NMP, MnSOD, and MMP. WX-132-18B and the three MIAs increased NMP and reduced MnSOD and MMP in a concentration-dependent manner; they displayed similar hepatotoxic phenotype profiles at the cytotoxic concentration. WX-132-18B is similar to colchicine, and exhibits a stronger impact on the redox system and mitochondrial function, but a weaker impact on NMP increase, than do taxol or vincristine. This further confirmed the nature of the mechanism of action of WX-132-18B. MnSOD expression is upregulated by oxidative stresses, including reactive oxygen species (ROS) increases, which are mainly due to mitochondrial and DNA damage [35]. The reduction of MnSOD content after 24 h treatment with WX-132-18B or MIA implied that mitochondrial damage may not be the initial drug target, but rather an accompanying event of cytotoxicity of these drugs; the initial microtubule inhibition has little or no effect on mitochondria, suggesting that mitochondrial dysfunction may not be the primary pathway in MIA apoptosis induction. The mechanism by which microtubule inhibitors inhibit the expression of MnSOD is currently not clear and is yet to be identified. Additionally, the effect of the compounds tested on NMP was efficient at lower concentrations, indicating that WX-132-18B and the three MIAs specifically alter cellular NMP and that NMP is closely related to microtubule function. Because enlargement of cell nuclear pores will allow more substances that induce cellular apoptosis to enter into the nucleus [36], this may be an additional mechanism by which WX-132-18B and the three MIAs promote cell apoptosis. In summary, the imbalance of redox system homeostasis and NMP augmentation constitute the primary cause of WX-132-18B and MIA cytotoxicity.

In addition, 22 fluorescence-tagged reporter cell lines directly or indirectly related to the pathways or targets of tumors, including AKT, PI3K, Raf, p38 MAPK, p53, and Rad51, were used to screen the possible impact of WX-132-18B on other potential targets (Supplementary Table 1). WX-132-18B exhibited results consistent with the three MIAs in this assay. They only slightly induced the formation of Rad51 foci in the U2OS cells at more than 30- to 100-fold concentrations and did not affect the other 21 targets or pathways. Rad51 foci are an indicator of cellular DNA damage [37]. Although at higher concentrations WX-132-18B and MIAs slightly damaged DNA, their maximal effects were only 25% of that induced by camptothecin, a specific inhibitor of type I topoisomerase [38]. These results indicate DNA damage is a non-specific toxic effect of these compounds and may be the result of NMP increase and apoptosis. These data further demonstrate that WX-132-18B has relatively good target selectivity.

High activity, anti-drug resistance, and tumor angiogenesis inhibition are three main aims in developing novel MIAs. Unlike the three marketed MIAs tested, WX-132-18B showed broad-spectrum anti-proliferative activity in eight different types of human cell lines, including six tumor cells, one taxol-resistance breast tumor cells, and HUVECs, with IC_50_ values ranging from 0.45 to 0.99 nM (far lower than those of taxol, colchicine and vincristine). Most importantly, an *in vivo* pharmacodynamics study revealed that WX-132-18B dose-dependently inhibited the growth of xenograft tumors in S180 sarcoma KM mice and H460 human non-small lung cancer and BGC-823 human gastric cancer-bearing nude mice, without affecting body weight during treatment. In correspondence with the *in vitro* result, the IHC assay of BGC-823 tumors showed that the number of tumor vessels strikingly decreased, and apoptotic cells increased dramatically. WX-132-18B had definite effects on cancer and tumor angiogenesis, although its anti-drug resistance *in vivo* still requires further study.

In summary, the investigation of tubulin binding and cellular phenotypic profiles of the cytoskeleton, cytotoxicity, and signal pathways demonstrated that WX-132-18B is a novel microtubule-depolymerizing agent that selectively binds to the colchicine-binding site of tubulin. Its potent anti-tumor activity, high selectivity, tumor vascular inhibition, and potential anti-resistance make WX-132-18B a promising anti-tumor candidate drug.

## MATERIALS AND METHODS

### Materials

Compound WX-132-18B (purity ≥98%) was designed and synthesized in our institute; the structure was validated using nuclear magnetic resonance/mass spectrometry (NMR/MS) and the purity was determined via high performance liquid chromatography (HPLC) [32]. Colchicine, vincristine, and taxol were purchased from Merck (Darmstadt, Germany). All the compounds were dissolved in dimethyl sulfoxide (DMSO) to a 10 mM stock and stored at −20°C.

TPCK-treated trypsin and bovine serum albumin were obtained from Sigma-Aldrich (Missouri, USA). Hoechst 33342, MitoTracker Red CMXRos, TOTO-3 Iodide, Alexa Flour 488 phalloidin; donkey anti-mouse Alexa Fluor 488 and Alexa Fluor 546 secondary antibodies; and mouse antibodies against MnSOD and α-tubulin were from Life Technologies (CA, USA). Rabbit antibodies against Ki67, Caspase 3, and CD31, horseradish peroxidase-labeled goat anti-rabbit secondary antibodies, the diaminobenzidine (DAB) substrate kits, and the HE staining kit were purchased from Wuhan Boster Biological Technology, Ltd (Wuhan, China). The Alexa Fluor 488 Annexin V/Dead Cell Apoptosis Kit was purchased from Invitrogen (Oregon, USA).

### Cell lines

Cell lines used for the signaling pathway screening (Supplementary Table 1) were purchased from GE Health Care (MA, USA) and Thermo Scientific (New Hampshire, USA), and cultured as per the product instructions. HepG2, HeLa, A549, H460, BGC-823, MX-1, MX-1/T, and HUVEC were purchased from the Basic Medical Center of the Institute of Basic Medical Sciences, Chinese Academy of Medical Sciences, and cultured in RPMI-1640 medium at 37°C in a humidified 5% CO_2_ incubator, except for the A549 cells, which were cultured in Ham's F12 medium. All media were supplied with 10% fetal bovine serum, and for MX-1/T cell line, 10 nM of taxol was added.

### Cell viability assay

The SRB assay was used to assess the anti-proliferative activities of the tested compounds [39]. Briefly, the cells (2.5 to 8.0 × 10^3^ cells/well, depending on cell type) were seeded into a 96-well plate and cultured overnight before exposure to the test compounds. After 72 h of compound treatment, the cells were fixed with trichloroacetic acid (TCA, 10%) at 4°C for 1 h, washed with double distilled water five times, and dried at 25°C (commonly defined as room temperature, RT) successively. The cells were then stained with SRB (0.4% in 1% acetic acid) at RT for 30 min and washed with 1% acetic acid five times, and dried at RT successively; 10 mM Tris base solution was then added, and the absorbance at 510 nm was measured. In each well plate, a vehicle control group and a positive drug control group were included. Each testing concentration included at least three repeat wells. The data was processed as described [40]. The anti-proliferative activities were expressed as the inhibitory rate. The inhibitory rate (%) = (A_control of 72h_ − A_compound of 72h_) / (A_control of 72h_ − A_control of 0h_) * 100, where A is the absorbance at 510 nm.

### Flow cytometry analysis

To detect cellular apoptosis induced by the tested compounds, the Alexa Fluor 488 Annexin V/Dead Cell Apoptosis Kit was used. A549 cells seeded in six-well plate at a density of 8 × 10^4^ cells/mL were cultured overnight and then treated with test compounds for 24 h before harvesting via centrifugation. The cells were stained with Annexin V and PI according to the manufacturer's instructions and measured using FACSCalibur Cytometer (BD Biosciences, CA, USA). Cells were classified as “survival”, “early apoptosis”, “late apoptosis”, and “necrosis” according to the extent of staining by Annexin V or/and PI.

For the cell cycle analysis, the collected cells were fixed in 70% ethanol in phosphate buffer saline (PBS) overnight at 4°C and treated with 0.05 mg/mL RNase and 3 μM PI successively before cell cycle detection. The cell cycle was divided into G0/G1, S, and G2/M phases, based on the extent of DNA staining.

### HCA multiparametric analysis

HCA multiparametric analysis was conducted as previously described [41]. For cytoskeleton multiparametric analysis, A549 cells seeded at a density of 8 × 10^3^ cells/well into a 96-well black plate (Costar 3603, USA) were cultured overnight and then treated with test compounds for 24 h. The cells were then fixed and cellular nuclei, actin, and microtubules were stained with Hoechst 33342, Alexa Flour 488 phalloidin, and α-tubulin antibody; the cells were then incubated with Alexa Flour 546 labeled secondary antibody. Incubation times were performed per the manufacturer's instructions.

For apoptosis detection, HepG2 cells cultured in a 96-well black plate were treated for 24 h. The cells were washed twice with Annexin V binding buffer (NaCl 140 M, HEPES 10 mM, CaCl_2_ 2.5 mM, pH 7.4), and labeled with Annexin V-Alexa and 3 μM PI for 15 min. Cells were then stained with 1 μM Hoechst 33342 in PBS for 1 h.

For cytotoxicity multiparametric analysis, HepG2 cells grown in a 96-well black plate were incubated with the compounds for 24 h. MitoTracker Red CMXRos, Hoechst 33342, and TOTO-3 Iodide were added to the cells before the cells were fixed to identify MMP, nuclei, and NMP, respectively. Cell MnSOD was then detected with primary antibody and Alexa Fluor 488-labeled secondary antibody.

For cellular signaling pathway screening, cells stably expressing a pathway-related protein fused with enhanced green fluorescence protein (EGFP) were used (Supplementary Table 1). When the signaling pathway is affected in response to the action of a tested compound, the change in the signaling protein can be detected via its expression level, puncta area, or translocation from cytosol to nucleus or membrane and vice versa. These changes can then be quantitatively analyzed. In this study, compounds were screened in two ways: the agonist mode, in which only the compound is added; and the antagonist mode, in which the compound is added simultaneously with an agonist. The reporter cells were seeded into 96-well black plates at an appropriate density and cultured overnight; the culture medium was then replaced by the corresponding assay buffer. The treatment duration and the concentration of positive drugs used on various cell lines were in accordance with the manufacturer's instructions (Supplementary Table 1). The cells were fixed and stained with 1 μM Hoechst 33342 in PBS for 2 h after treatment.

Finally, the staining solution was removed, cells were kept in PBS, and images were acquired in IN Cell Analyzer 2000 (GE HealthCare, USA) under a 20× objective lens for the assays. For all measurements, filters were set according to the wavelength of the fluorescent dyes, and nine fields from each well and not less than 200 cells were obtained. The acquired images were analyzed using an IN Cell Analyzer Workstation 3.5 and the Multi Target Analysis Module (GE Health Care, USA). The final measurements were averaged over the population of cells detected in the fields of view. The output parameters and their implications are shown in Table 2. The other detection and assay conditions are the same as described in our previous paper [41]. In all experiments, cell culture medium containing 0.2% DMSO was designated as the blank control, and was included in each plate to allow data normalization and plate quality control. Each test was performed in triplicate.

### Competitive tubulin-binding assay

For evaluation of the colchicine-binding site on tubulin, 4 μM of tubulin prepared from fresh dog brain tissue [42, 43] was added to PEM buffer (25 mM PIPES, 1 mM EGTA, 3 mM MgCl_2_, pH 6.8) and incubated with or without various concentrations (0.1, 0.3, 1, 2, 3, and 10 μM) of WX-132-18B or vincristine at 37°C for 45 min in a nontransparent black 96-well plate. Then, 10 μM of colchicine was added to the mixture and incubated at 37°C for 45 min. The fluorescence intensity of the reaction system (ex. 340 nm, em. 435 nm) was measured. The inhibition rate was expressed as the percentage (%) of decreased fluorescence of the tubulin−colchicine complex and calculated using the following formula: inhibition rate % (IR) = (I_p_−I_c_) / (I_p_− I_n_) * 100%, where I is the fluorescent intensity, “p” and “n” denote positive control (10 μM colchicine) and negative control (blank control solution without colchicine), respectively. “c” represents the tested compound solution with tubulin. Vincristine, which cannot bind to the colchicine-site of tubulin, was added as a negative control.

To evaluate the vinblastine-binding site on tubulin, tubulin (4 μM) in PEM buffer was mixed with WX-132-18B, colchicine, and vincristine at concentrations of 0.01, 0.1, 1, 3, 10, 30, and 100 μM and incubated at 37°C for 45 min. BODIPY FL-vinblastine (2 μM) was then added, and the mixtures were incubated for 45 min. The fluorescence intensity of the FL-vinblastine-tubulin complex (ex. 470 nm, em. 515 nm) was measured. The reduction in the fluorescence intensity of the tubulin-BODIPY FL-vinblastine complex was measured and converted into inhibition rates. Inhibition rate % (IR) = (I_p_−I_c_)/(I_p_− I_n_) * 100%, where “p” and “n” denote positive control (2 μM FL-vinblastine) and negative control (blank control solution without tubulin), respectively.

### Limited proteolysis assay

Tubulin (finial concentration 1 mg/mL) was mixed with PEM buffer as mentioned above, as well as 10 μM colchicine, 10 μM vincristine, or 10 μM WX-132-18B, and incubated at 30°C for 30 min. TPCK-treated trypsin was then added (1:40, w/w to tubulin), and the reaction system was incubated on ice in the dark for 20 min. The samples were mixed with 4× SDS loading buffer and boiled for 5 min before resolution via a 10% SDS-PAGE gel. The gel was stained with Coomassie Brilliant Blue, and the image was acquired using the gel imaging system Chemi Imager 5500 (Alpha Innotech, CA, USA).

### *In vivo* antitumor efficacy study

Approximately 6 × 10^6^ S180 cells were injected subcutaneously into the right axilla of six- to eight-week-old KM mice (purchased from the Experimental Animal Center of the Academy of Military Medical Sciences, Beijing, China). Seven to 10 days after tumor cell implantation, all mice were randomly divided into treatment groups and a control group (n=10) and treated with WX-132-18B (1.0 mg/kg, 0.5 mg/kg, or 0.25 mg/kg) or vehicle (saline) by intraperitoneal (i.p.) injection twice a week for three weeks.

Approximately 6 × 10^6^ H460 cells or BGC-823 cells were injected subcutaneously into the right axilla of eight-week-old nude mice (obtained from Beijing Vital River Laboratory Animal Technology Co., Ltd). When the tumor grew to 2 to 3 cm in diameter, the tumor blocks were removed and divided into tiny blocks about 2.5 mm^3^ and transplanted subcutaneously to the mice in sterile conditions. Seven to 10 days after the implantation, all mice were randomly divided into control, taxol (15 mg/kg), and WX-132-18B (1.0 mg/kg, 0.5 mg/kg or 0.25 mg/kg) treatment groups (n=8). Drugs were given once every five days to H460 transplanted nude mice and twice a week to BGC-823 transplanted nude mice via i.p. injection for three weeks.

Tumor growth during treatment was measured with a slide caliper, and volumes were estimated according to the following formula: tumor volume (mm^3^) = *L* × *W*^2^ × 0.5, where *L* is length and *W* is width. After all the experiments were completed, the mice were killed and tumor weights measured. All animal experiments were conducted strictly in accordance with the guidelines of the Animal Experimentation Ethics Committee of Beijing Institute of Pharmacology and Toxicology for animal care, handling, and termination.

### Histological assay

At the end of the treatment period, the tumor tissues were dissected from the mice, fixed in 4% paraformaldehyde, and embedded in paraffin, and tissue sections (4 mm) were prepared [44]. Sections were then cut at a thickness of 4 μm and stained with CD31, Caspase 3, or Ki 67. The immunohistochemistry reaction was developed with a DAB substrate kit prior to counterstaining the slides with hematoxylin and eosin (H&E).

### Data processing and statistical analysis

Data analysis for all HCA were conducted as reported previously [41]. All data from the *in vitro* experiments were expressed as means ±*SD* of three independent experiments. IC_50_ values were calculated with Origin 6.1 software. Statistical analyses were performed using one-way analysis of variance (ANOVA) with SPSS 11.5 software (*P<0.05, **P<0.01, ***P<0.001).

## SUPPLEMENTARY MATERIALS FIGURES AND TABLES





## Abbreviations

MIA: microtubule-inhibiting agent
HCA: high content assay
SRB: sulforhodamine B
HUVEC: human umbilical vein endothelial cells
TPCK: tosyl-phenylalanine chloromethyl-ketone
FCM: flow cytometry
I: fluorescent intensity
A: fluorescent puncta area
MMP: mitochondrial membrane potential
MnSOD: manganese superoxide dismutase
NMP: nuclear membrane permeability
KM: Kunming
HE: hematoxylin and eosin
IHC: immunohistochemistry
HCI: high content image
ROS: reactive oxygen species
NMR: nuclear magnetic resonance
MS: mass spectrometry
HPLC: high performance liquid chromatography
DAB: diaminobenzidine
TCA: trichloroacetic acid
RT: room temperature
PBS: phosphate buffer saline
EGFP: enhanced green fluorescence protein
PIPES: Piperazine-1,4-bisethanesulfonic acid
EGTA: ethylenebis (oxyethylenenitrilo) tetraacetic acid
HEPES: 4-(2-hydroxyerhyl) piperazine-1-erhanesulfonic acid
DMSO: dimethyl sulfoxide
IR: inhibition rate
i.p.: intraperitoneal
