# Supplementary material for: WX-132-18B, a novel microtubule inhibitor, exhibits promising anti-tumor effects
Source: Oncotarget. 2017 May 9;8(42):71782–96. doi: 10.18632/oncotarget.17710 (PMC5641089; doi:10.18632/oncotarget.17710)
Supplement: Supplementary file 1 [file oncotarget-08-71782-s001.pdf]

WX-132-18B, a novel microtubule inhibitor, exhibits promising anti-tumor effects

SUPPLEMENTARY MATERIALS

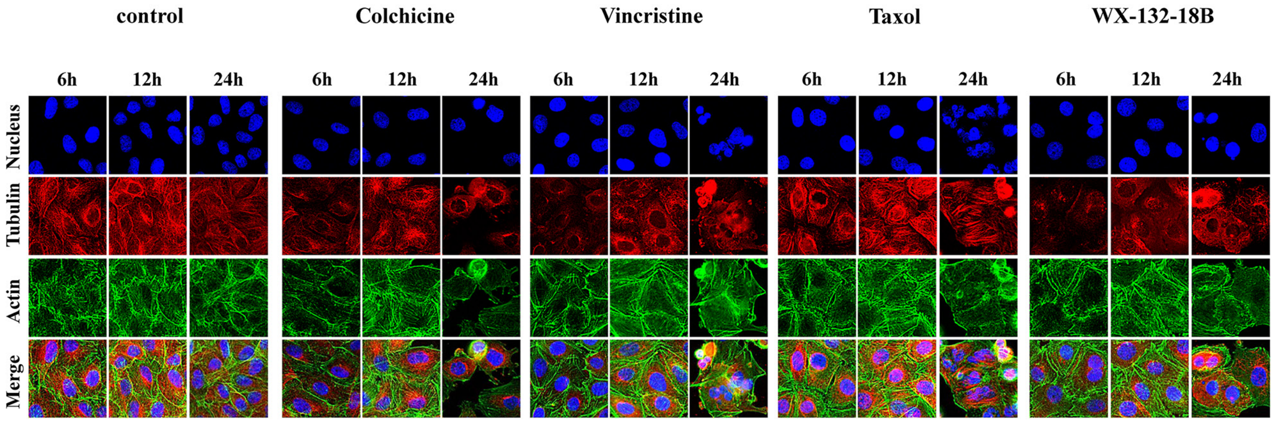

Supplementary Figure 1: Changes of cytoskeleton after treatment with compound WX-132-18B for 6 h, 12 h, or 24 h. A549 cells were treated with vehicle (0.1% dimethyl sulfoxide), colchicine (100 nM), vincristine (100 nM), taxol (100 nM), or WX-132-18B (3 nM).

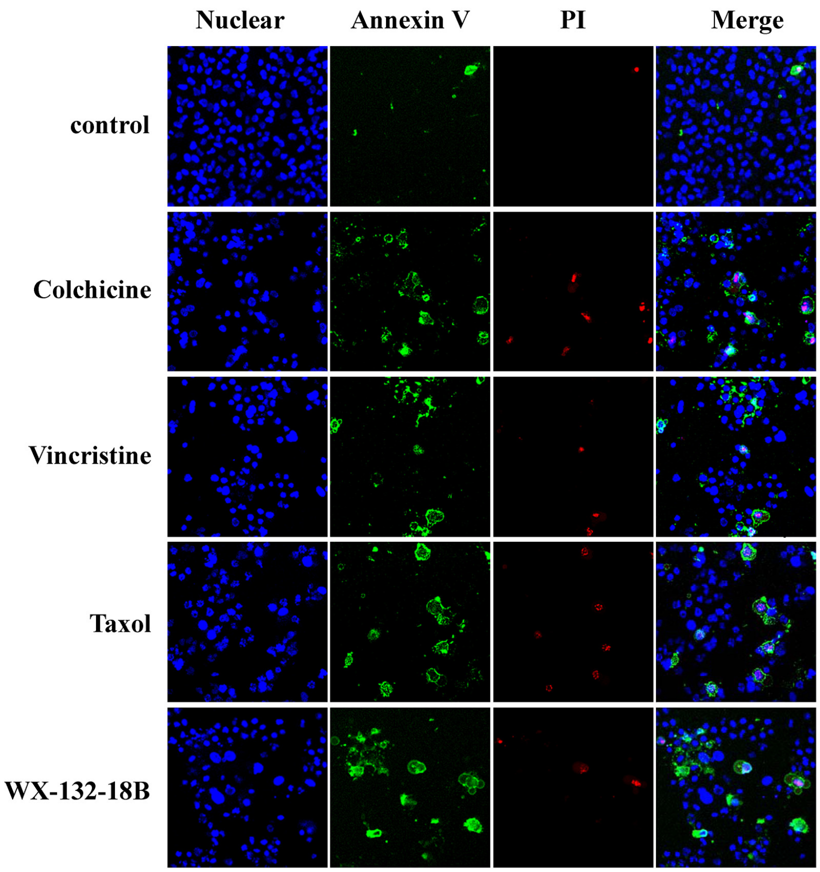

Supplementary Figure 2: Effect of compound WX-132-18B on apoptosis in A549 cells. Cells were treated with 300 nM colchicine, 300 nM vincristine, 100 nM taxol, or 3 nM WX-132-18B for 24 h. A triparametric cytofluorometric assay using propidium iodide (PI) to stain DNA (red), AnnexinV-Alexa Fluor 488 to label phosphatidylserine (green), and Hoechst 33342 to label nuclei (blue) was performed. Images were acquired with IN Cell Analyzer 1000 using a 20× objective lens. Cells exhibiting red fluorescence were those undergoing necrosis, green fluorescence were those undergoing early apoptosis, and a mix of the two colors were classified as those undergoing late apoptosis.

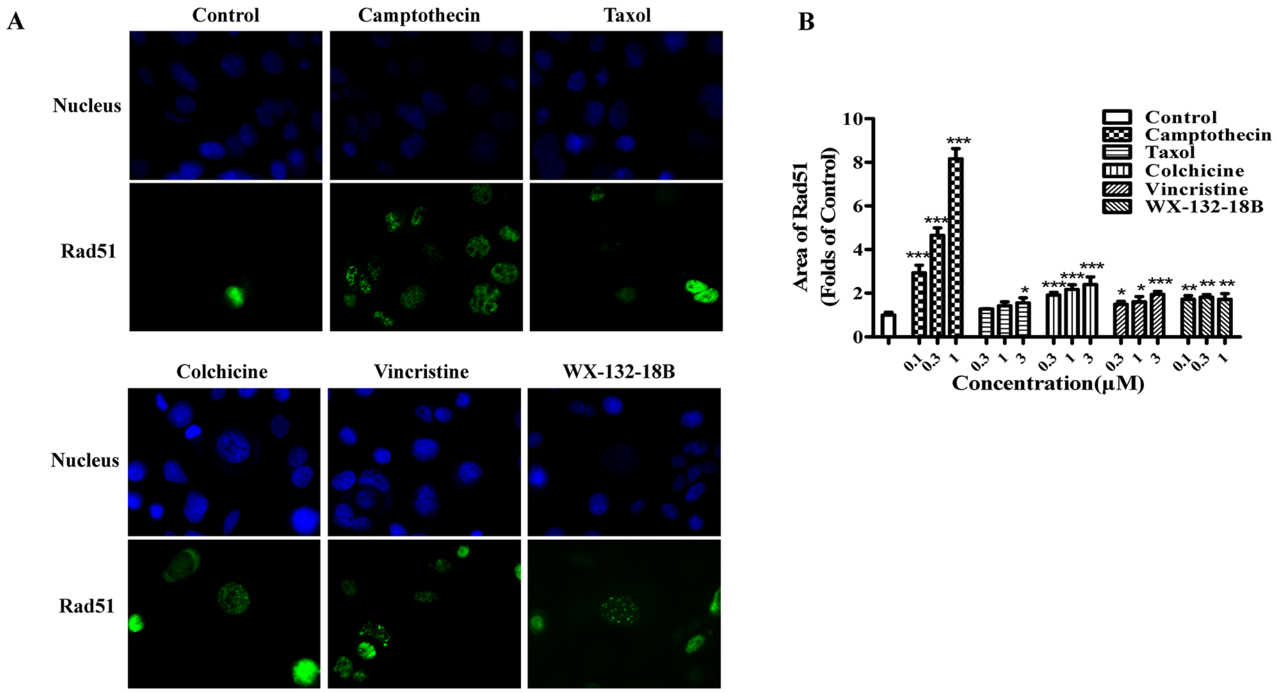

Supplementary Figure 3: Effect of compound WX-132-18B on the formation of Rad51 foci in Rad51-EGFP-U2OS cells. **(A)** The accumulation of Rad51 in Rad51-EGFP-U2OS cells treated with vehicle (0.1% dimethyl sulfoxide), camptothecin (1 μM), taxol (1 μM), colchicine (1 μM), vincristine (3 μM), and WX-132-18B (1 μM) for 24 h. **(B)** Quantification of Rad51 foci area in different groups. Values are mean±SD, n=3. *P<0.05, **P<0.01, ***P<0.001, compared with control group.

Supplementary Table 1:

See Supplementary File 1
